# Survival Comparisons between Breast Conservation Surgery and Mastectomy Followed by Postoperative Radiotherapy in Stage I–III Breast Cancer Patients: Analysis of the Surveillance, Epidemiology, and End Results (Seer) Program Database

**DOI:** 10.3390/curroncol29080452

**Published:** 2022-08-15

**Authors:** Wenbin Xiang, Chaoyan Wu, Huachao Wu, Sha Fang, Nuomin Liu, Haijun Yu

**Affiliations:** 1 Hubei Cancer Clinical Study Center, Zhongnan Hospital of Wuhan University, Wuhan 430071, China; 2 Department of Radiation and Medical Oncology, Zhongnan Hospital of Wuhan University, Wuhan 430071, China; 3 Hubei Key Laboratory of Tumor Biological Behaviors, Wuhan 430071, China; 4 Department of Integrated Traditional Chinese Medicine and Western Medicine, Zhongnan Hospital of Wuhan University, Wuhan 430071, China

**Keywords:** breast cancer, SEER, breast-conserving surgery plus radiotherapy, mastectomy plus radiotherapy, prognostic nomogram

## Abstract

Background: This study aims to evaluate the overall and breast cancer-specific survival (BCSS) after breast-conserving surgery (BCS) plus radiotherapy (RT) compared with mastectomy plus RT in resectable breast cancer. Moreover, the aim is to also identify the subgroups who benefit from BCS plus RT and establish a predictive nomogram for stage II patients. Methods: Stage I–III breast cancer patients were identified from the Surveillance, Epidemiology, and End Results (SEER) database between 1990 and 2016. Patients with available clinical information were split into two groups: BCS plus RT and mastectomy plus RT. Kaplan–Meier survival analysis, univariate and multivariate regression analysis, and propensity score matching were used in the study. Hazard ratio (HR) was calculated based on stratified Cox univariate regression analyses. A prognostic nomogram by multivariable Cox regression model was developed for stage II patients, and consistency index (C-index) and calibration curve were used to evaluate the accuracy of the nomogram in the training and validation set. Results: A total of 24,590 eligible patients were enrolled. The difference in overall survival (OS) and BCSS remained significant in stage II patients both before and after PSM (after PSM: OS: HR = 0.8536, *p* = 0.0115; BCSS: HR = 0.7803, *p* = 0.0013). In stage II patients, the survival advantage effect of BCS plus RT on OS and BCSS was observed in the following subgroups: any age, smaller tumor size (<1 cm), stage IIA (T2N0, T0–1N1), ER (+), and any PR status. Secondly, the C-indexes for BCSS prediction was 0.714 (95% CI 0.694–0.734). The calibration curves showed perfect agreement in both the training and validation sets. **Conclusions:** BCS plus RT significantly improved the survival rates for patients of stage IIA (T2N0, T0–1N1), ER (+). For stage II patients, the nomogram was a good predictor of 5-, 10-, and 15-year BCSS. Our study may help guide treatment decisions and prolong the survival of stage II breast cancer patients.

## 1. Background

Breast-conserving surgery (BCS) plus postoperative radiotherapy (RT) was recommended as an alternative to mastectomy for early-stage breast cancer patients based on several randomized controlled trials, demonstrating that BCS followed by postoperative RT is as effective as mastectomy [1,2,3,4]. Recently, data from several population-based studies suggest the superiority of BCS plus RT over mastectomy without RT [5,6,7,8,9,10,11]. With the advancements and standardization of surgery and postoperative adjuvant RT technology, stage II and a considerable proportion of stage III patients tend to receive BCS [9,10,11]. European and American guidelines recommend RT after mastectomy for patients with stage T3, extensive tumor multifocality, and positive axillary lymph nodes [12,13,14,15,16,17]. However, no randomized controlled trials investigated the survival outcomes between BCS plus RT group and mastectomy plus RT group. The investigation of survival differences based on a large sample database between BCS plus RT and mastectomy plus RT and of the people who benefit more from BCS plus RT are, therefore, of interest.

Veronesi U. et al. conducted a randomized trial in 1973, demonstrating the non-inferiority of BCS followed by RT compared with radical (Halsted) mastectomy for patients with a tumor size of 2 cm or smaller [1]. At the same time, a randomized controlled trial conducted by Fisher B. et al. confirmed the finding [2]. Therefore, BCS plus RT was recommended as an alternative to mastectomy for early-stage breast cancer patients [18]. Many recent studies (e.g., Hwang ES, 2013; Agarwal S, 2013; Fisher S, 2015; Hartmann-Johnsen OJ, 2015) have shown the superiority of BCS plus RT over mastectomy without RT [5,6,7,8,9,10,11]. However, fewer studies investigated whether BCS plus RT was still superior to mastectomy in the case of RT. Agarwal’s study demonstrated that female patients undergoing BCS plus RT have a better 5-year and 10-year disease-specific survival rate compared with those who received mastectomy with or without RT, and the number of the patients who underwent mastectomy plus RT accounted for 3% [6]. Moreover, Hartmann-Johnsen OJ’s study to compare the survival of BCS plus RT and mastectomy analyzed the Norwegian population of T1–2N0–1M0 from 1998 to 2008 and reported a worse disease-specific survival in the mastectomy group (30.7% of patients of the mastectomy group received postoperative RT) [8]. These studies showed that mastectomy plus RT seemed to be associated with worse survival compared with the BCS plus RT group. In Lan XW’s retrospective study of 196 pairs of stage T1–2N1M0 Chinese female patients, a lower 5-year distant metastasis rate and superior 5-year distant metastasis-free survival and disease-free survival and breast-cancer-specific survival (BCSS) with receipt of BCS plus RT compared with mastectomy plus RT were reported [19]. On the contrary, Sun GY et al. compared 244 pairs of stage T1–2N1M0 Chinese patients, showing that the BCS plus RT group had comparable survival outcomes to the mastectomy plus RT group [20]. According to de Boniface J’s analysis of Swedish female patients with invasive T1–2 N0–2 breast cancer from 2008 to 2017, BCS plus RT has a superior 5-year overall survival (OS) and 5-year BCSS than mastectomy with or without RT [21].

Here, we extracted patient-level data from the Surveillance, Epidemiology, and End Results (SEER) database and divided it into two cohorts: BCS plus RT and mastectomy plus RT. The study included all stage I–III female patients with breast cancer from 1990 to 2016. Not only early-stage patients but also T3–4 (tumor size > 5 cm) and N2–3 (four or more node metastases) patients were analyzed in our study.

Our population-based study aimed to compare long-term survival by analyzing large samples. Propensity score matching (PSM) was used to balance the deviation of confounding factors. Furthermore, for stage II patients, we conducted a subgroup analysis based on multiple independent prognostic factors to explore specific beneficiaries. Lastly, a predictive nomogram was established for stage II patients.

## 2. Methods and Materials

### 2.1. Data Sources

The SEER database consists of several tumor registries in different regions, collecting information about 26% to 30% of the US population. It contains detailed demographic, socioeconomic, cancer, and treatment information. The SEER data is publicly available. We obtained the clinical data of female patients diagnosed with breast cancer in the SEER database from 1990 to 2016 (November 2018 Submission, SEER 13). The data includes age at diagnosis, race, pathological type, grade, laterality, TNM stage, tumor size, lymph node infiltration, estrogen receptor (ER) status, progesterone receptor (PR) status, treatments, and follow-up data.

### 2.2. Cohort Selection

We used SEER *-Stat 8.3.8 software (National Cancer Institute, Bethesda, MD, USA) to extract data from the SEER database. Patients diagnosed with primary breast cancer (SEER cancer site code: 50.0) in American Joint Committee on Cancer (AJCC) stage I to III from 1990 to 2016 were included in our study. Patients who had previously undergone BCS (site-specific surgery codes 20–24) plus RT or mastectomy (site-specific surgery codes 30, 40, 50, 60, 70, 76) plus RT were screened out. Patients with complete clinical and demographic information were included. Using the histological code of ICD-0–3, the patients were divided into lobular carcinoma (8570 to 8580), ductal carcinoma (8500), and other pathological types. The 7th edition of AJCC breast cancer was released in 2009, so the patients diagnosed between 1990 and 2010 were staged with 6 editions, and the patients after 2010 were staged with 7 editions. To eliminate deviations due to different RT techniques, patients only receiving external beam radiation therapy were included. The following patients were excluded: those who lack clinical and follow-up information and those whose surgical methods are unknown (site-specific surgery codes 99, site-specific surgery codes 90).

### 2.3. Variables

The data extracted in this study included age, race, pathologic grade, laterality, stage, tumor size, lymph node infiltration, ER status, PR status, chemotherapy, and treatment strategies (BCS plus RT vs. mastectomy plus RT). The study focused on OS, BCSS, and hazard ratio (HR). OS refers to the time a patient lived from breast cancer diagnosis to death from any cause, and BCSS refers to deaths from breast cancer.

### 2.4. Propensity Score Matching

Propensity score matching (PSM), proposed in 1983 by Rosenbaum and Rubin, and not limited by the number of events, was performed for all relevant confounding factors to minimize selection bias [22,23]. The nearest available neighbor matching and caliper matching were used in our research in PSM [24,25]. For the patients of stage III, the ratio of PSM is 1:1. For the patients of stage II, to balance the effects of confounding, PSM with a 1:1 ratio and the caliper of 0.02 was set. Only when the propensity score of the control group (mastectomy plus RT) is within a certain distance (0.02), the control group will be matched with the case. Matched covariates include age, race, stage, tumor size, ER and PR status, and chemotherapy.

### 2.5. Statistical Methods

In this study, all data are counted by EXCEL, and differences in baseline characteristics between the two groups of people were assessed by the χ2 test or Fisher exact test (BCS plus RT vs. mastectomy plus RT). The potential risk factors of OS and BCSS were analyzed by the univariate and multivariate Cox regression model; 95% confidence interval and log-rank test were calculated. The 5-year, 10-year, and 15-year OS and BCSS were assessed with the Kaplan–Meier method and compared with the log-rank test. The results of the subgroup analysis were presented by the forest plot. The nomogram construction and validation were performed based on the results of the Cox proportional risk model. Receiver operating characteristic (ROC) and calibration curve were used to evaluate the accuracy of the model. The C-index value is positively correlated with the predictive performance of the model. Calibration curves were plotted at 5, 10, and 15 years by a bootstract involving 100 resamples. Ideally, the points on the calibration diagram should be close to the 45° diagonal. The above statistical analysis was completed by R software (version 4.2.0, Vienna, Austria), using R packages such as “RMS”, “Foreign”, “Survival”, “forestplot”, “Tableone”, “MatchIt”, “survivalROC”, “caret” etc. *p* < 0.05 is regarded as the statistical difference.

## 3. Results

### 3.1. Patient Characteristics and Determining Independent Prognostic Factors

**Figure 1:**
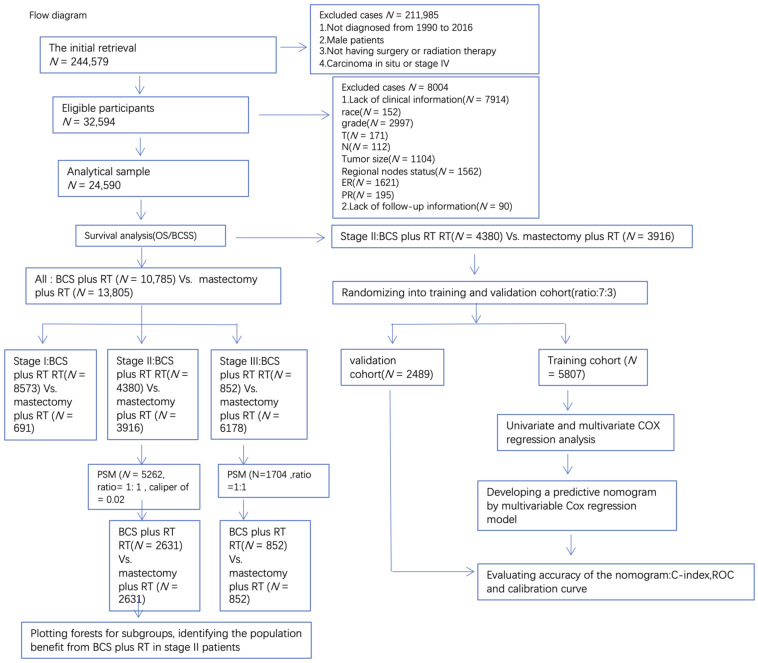
Flow diagram of selecting eligible patients.

The data filtering process is shown in Figure 1. A total of 24,590 eligible patients were enrolled, including mastectomy plus RT (10,785, 43.9%) vs. BCS plus RT (13,805, 56.1%). Our data indicated that older patients (>65), better differentiated (grade I/II), earlier staged (stage I), T1, N0, smaller size (<2 cm), and ER and PR positive patients are more likely to receive BCS plus RT, which is consistent with most clinical guidelines (Table 1). The results of multivariate regression were consistent with the results of univariate regression. The results showed that all factors affected the Stage I–III breast cancer patients’ OS and BCSS except laterality and histologic type (Supplementary Tables S1 and S2). It is worth mentioning that treatment significantly affected the OS and BCSS of Stage I–III patients. BCS plus RT can reduce the risk of death by 15% (HR = 0.8473 (CI 0.7841–0.9155) *p* < 0.001. Supplementary Table S1) and the risk of specific death from breast cancer by 18% (HR = 0.820 (CI 0.746–0.901) *p* < 0.001. Supplementary Table S2).

### 3.2. BCS Plus RT Improved Survival of Stage II Patients before and after PSM

**Figure 2:**
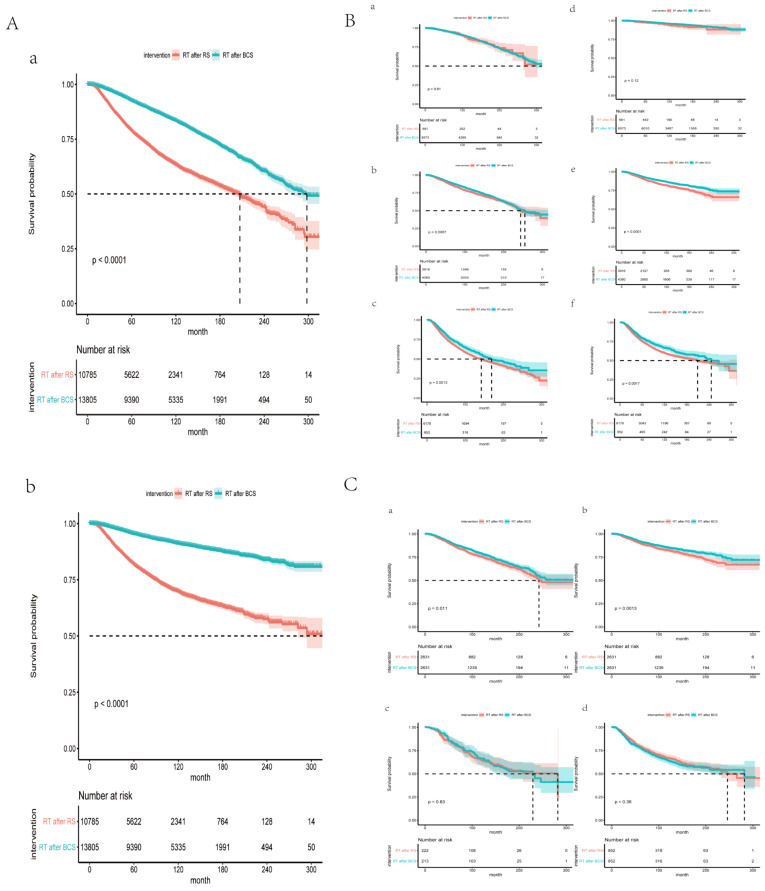
Survival analysis. (**A**) Meier survival curve of all breast cancer patients. (**a**) Meier survival curve on OS, (**b**) Meier survival curve on BCSS. (**B**) Survival curve of stage I, II, and III patients before PSM on OS and BCSS. (**a**) Meier survival curve of stage I breast patients before PSM for OS, *p* = 0.91. (**b**) Meier survival curve of stage II breast patients before PSM for OS, *p* = 0.0067. (**c**) Meier survival curve of stage III breast patients before PSM for OS, *p* = 0.0013. (**d**) Meier survival curve of stage I breast patients before PSM for BCSS, *p* = 0.12. (**e**) Meier survival curve of stage II breast patients before PSM for BCSS, *p* < 0.0001. (**f**) Meier survival curve of stage III breast patients before PSM for BCSS, *p* = 0.0017. (**C**) Survival curve of stage II and III breast patients after PSM on OS and BCSS. (**a**) Meier survival curve of stage II breast patients after PSM for OS, *p* = 0.011. (**b**) Meier survival curve of stage II breast patients after PSM for BCSS, *p* = 0.0013. (**c**) Meier survival curve of stage III breast patients after PSM for OS, *p* = 0.83. (**d**) Meier survival curve of stage III breast patients after PSM for BCSS, *p* = 0.36.

As shown in Figure 2A, patients who underwent BCS plus RT had better OS and BCSS than those who underwent mastectomy plus RT (*p* < 0.001). As discussed, our findings at least suggest that stage was an independent prognostic factor in all patients. Consequently, grouping by stage, survival comparison was explored in the BCS plus RT group and mastectomy plus RT group (Figure 2B). Log-rank tests indicated significantly different survival curves at the 5- year points 10-year points, and 15-year points (Stage II HR = 0.8712 (CI 0.7885–0.9625) *p* = 0.0067; Stage III HR = 0.8211 (CI 0.7281–0.9267) *p* = 0.0013). For stage I patients, there was no statistically significant difference (Stage I HR = 1.014 (CI 0.8005–1.285) *p* = 0.91). The 5-year, 10-year, or 15-year survival rates of BCS plus RT were higher than those of mastectomy plus RT for stage II-III patients. For stage II patients, the 5-year survival rate for patients who received BCS plus RT and mastectomy plus RT were 89.9% (95% CI 88.9–90.9%) and 87.7% (95% CI 86.5–88.9%), respectively. The 10-year survival rate for patients who received BCS plus RT and mastectomy plus RT were 79.1% (95% CI 77.5–80.7%) and 75.7% (95% CI 73.9–77.5%), respectively. The 15-year survival rate for patients who received BCS plus RT and mastectomy plus RT were 67.4% (95% CI 65.2–69.6%) and 65.6% (95% CI 62.9–68.3%), respectively. For stage III patients, the 5-year survival rate for patients who received BCS plus RT and mastectomy plus RT were 76.5% (95% CI 73.4–79.6%) and 71.7% (95% CI 70.5–72.9%), respectively. The 10-year survival rate for patients who received BCS plus RT and mastectomy plus RT were 60.2% (95% CI 56.3–64.1%) and 53.8% (95% CI 52.2–55.4%), respectively. The 15-year survival rate for patients who received BCS plus RT and mastectomy plus RT were 48.3% (95% CI 43.4–53.2%) and 44% (95% CI 42–46%), respectively.

To explore the specific beneficiaries of BCS plus RT, the patients were stratified into two different risk groups (stage II/III) for further evaluation. The χ2 test or Fisher exact test was used to compare the clinical characteristics between BCS plus RT and mastectomy plus RT. The results showed low comparability of the multiple factors for stage II or stage III patients (Table 2 and Table 3). PSM was conducted for stage II and III patients to eliminate the influence of all relevant factors, respectively (Table 2 and Table 3). As shown in Figure 2C, Kaplan–Meier analysis of adjusted stage II patients demonstrates that BCS plus RT still benefits the OS and BCSS compared with mastectomy plus RT (OS HR = 0.8536 (CI 0.755–0.9651), *p* = 0.0115; BCSS: HR = 0.7803 (CI 0.6706–0.9081) *p* = 0.0013). However, there was no statistically significant difference in the group of stage III patients (OS HR = 0.9103 (CI 0.7783–1.065) *p* = 0.2412; BCSS: HR = 1.086 (CI 0.9105–1.295) *p* = 0.3597).

### 3.3. Subgroups Benefiting from BCS plus RT in Stage II

**Figure 3:**
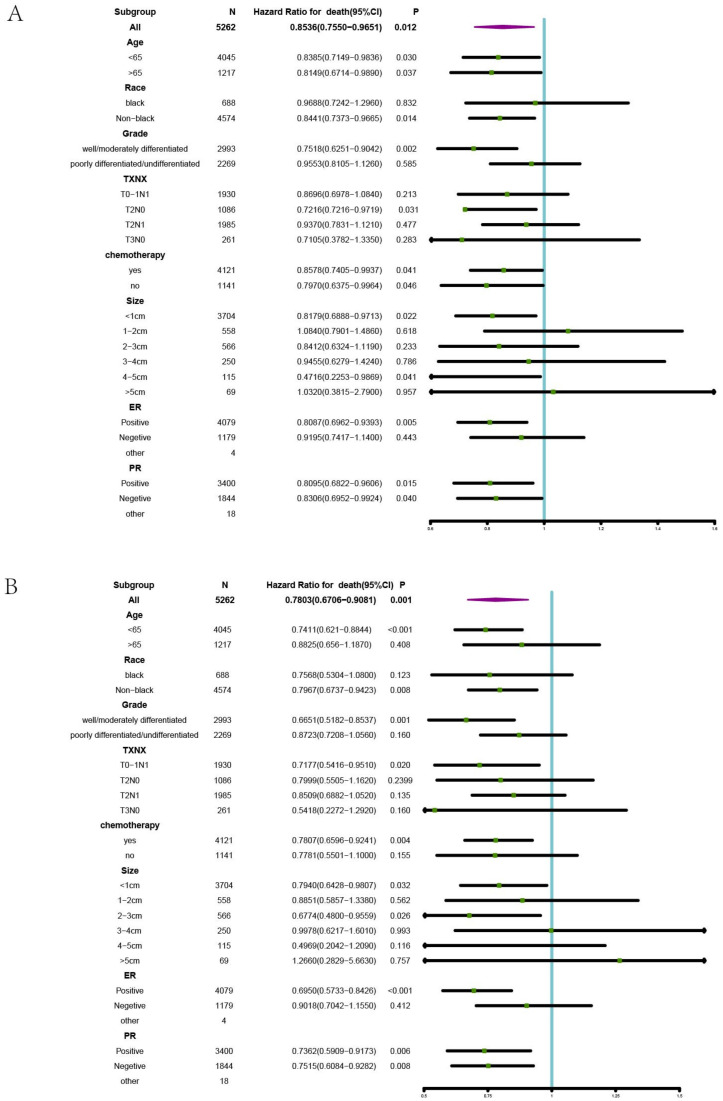
Subgroup analysis for OS and BCSS for stage II patients. (**A**) Forest map for OS (**B**) Forest map for BCSS.

**Figure 4:**
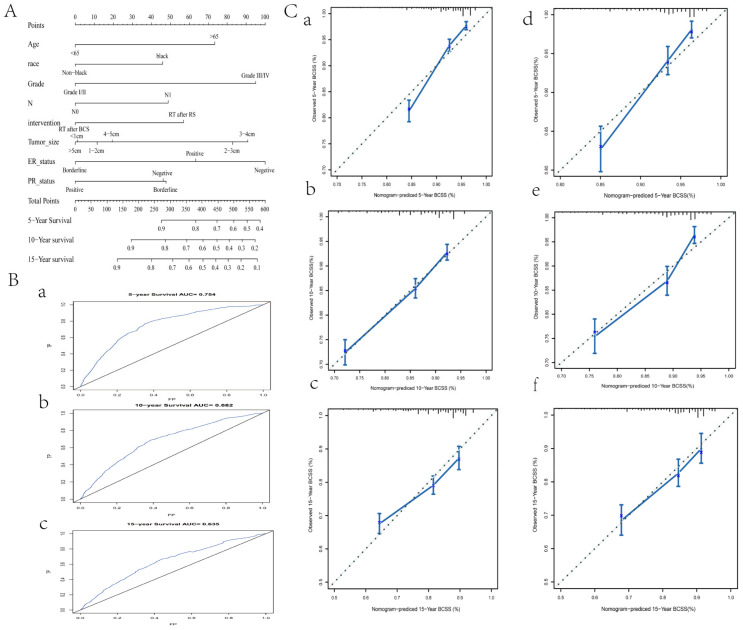
Prediction and validation of the nomogram in stage II patients. (**A**) The nomogram for the 5-, 10-, and 15-year BCSS prediction of stage II patients. (**B**) ROC curves verified the predictive value of the nomogram. (**a**) 5-year ROC curves. (**b**) 10-year ROC curves. (**c**) 15-year ROC curves. (**C**) Calibration of curves of 5, 10, and 15 years in the training set. (**a**) 5-year BCSS in the training set. (**b**) 10-year BCSS in the training set. (**c**) 15-year BCSS in the training set. (**d**) 5-year BCSS in the test set. (**e**) 10-year BCSS in the test set. (**f**) 15-year BCSS in the test set.

Furthermore, 8296 stage II cases were divided into the training set and test set in a ratio of 7:3. Univariate and multivariate analyses were conducted for stage II patients based on the training set. As shown in Table 4, age, race, grade, N, intervention, tumor size, ER status, and PR status were found to be independent prognostic factors for Stage II patients. Stratified Cox univariate regression analyses were performed for stage II patients, and the results were presented by forest plot (Figure 3). The survival advantage effect of BCS plus RT on OS was observed in all age subgroups compared with mastectomy plus RT (<65 HR = 0.8385 *p* = 0.030; >65 HR = 0.8149 *p* = 0.037). However, the effect of BCS plus RT on BCSS was observed only in a subset of patients younger than 65 years (<65 HR = 0.7411 *p* < 0.001; > 65 HR = 0.8825 *p* = 0.408). The OS and BCSS benefit with BCS plus RT was observed in the following four subsets: non-blacks group, well-differentiated group, tumor size < 1 cm, and ER(+) group (non-blacks: OS: HR = 0.8441 *p* = 0.014; BCSS: HR = 0.7967 *p* = 0.008; well-differentiated: OS: HR = 0.7518 *p* = 0.002; BCSS: HR = 0.6651 *p* = 0.001; < 1 cm: OS HR = 0.8179 *p* = 0.022; BCSS: HR = 0.7940 *p* = 0.032; ER (+): OS: HR = 0.8095 *p* = 0.015; BCSS: 0.6950 *p* < 0.001). In a subset of patients with T2N0 stage disease, the effect of BCS plus RT on OS was observed (OS: HR = 0.7216 *p* = 0.031), and the effect of BCS plus RT on BCSS was observed in the T0−1N1 subgroup (HR = 0.7177 *p* = 0.020). Regardless of receiving chemotherapy, the effect of BCS plus RT on OS can be observed (Yes: HR = 0.8578 *p* = 0041; NO: HR = 0.7970 *p* = 0046). However, only among patients receiving chemotherapy those who received BCS plus RT can benefit from BCSS (HR = 0.7807 *p* = 0.004). Regardless of PR status, the effect of BCS plus RT on OS and BCSS can be observed. Stratified Cox univariate regression analyses were performed for stage III patients, and the results were presented by forest plot (Figure 4). As shown in Supplementary Figure S1, in stage III patients, the survival advantage effect of BCS plus RT on OS and BCSS was observed in a subset of black (OS: HR = 0.6898 (0.5132–0.9273) *p* = 0.0375; BCSS: HR = 0.6222 (0.4475–0.8649) *p* = 0.0164).

### 3.4. A Predictive Nomogram for BCSS Based on Stage II Data

Based on these studies, BCS plus RT may lead to more prolonged survival, especially for stage II breast cancer patients. Age, race, grade, N, intervention, tumor size, ER status, and PR status were found to be independent prognostic factors of stage II patients. Based on the above factors, we plotted a predictive nomogram basing the results of multivariate Cox regression analysis and displayed the 5-, 10-, and 15-year BCSS probabilities (Figure 4A). The C-index for BCSS prediction was 0.714 (95% CI 0.694–0.734). Additionally, the ROC curves (Figure 4B) and the calibration curves of 5, 10, and 15 years all showed excellent predictive power of the model. The result of the validation set was consistent with that of the training set (Figure 4C).

## 4. Discussion

In this study, our results indicate that stage II-III breast cancer patients who underwent BCS plus RT had better OS and BCSS than those who underwent mastectomy plus RT, with no significant difference for stage I breast cancer patients. After adjusting for confounders by PSM, the difference in OS and BCSS remained significant in patients with stage II. In a subset of stage II breast cancer, the survival advantage effect of BCS plus RT on OS and BCSS was observed regardless of PR status. The survival advantage effect of BCS plus RT on OS was observed in the subset: all age, non-blacks, well-differentiation, T2N0, 1Cm, and ER-positive. Additionally, the BCS plus RT group younger than 65, well-differentiated, T0–1N1, <1 cm, 2–3 cm, and ER-positive subtypes can significantly benefit from BCSS rather than the mastectomy plus RT group, and the difference was statistically significant (Figure 3).

For female patients with early-stage breast cancer, randomized clinical trials and recent retrospective investigations have demonstrated that BCS followed by RT is not inferior to mastectomy and may even be superior [1,2,3,4,5,6,7,8,9,10,11]. Yet, fewer studies have shown whether BCS plus RT still surpassed mastectomy in the case of RT. Our findings were similar to those of Agarwal S et al. [6], which reported that when the number of lymph nodes was less than three, BCS plus RT was preferable to mastectomy and mastectomy plus RT. However, patients with three or more positive lymph node metastasis were excluded from their study, and the number of cases of mastectomy plus RT was small, accounting for only 3% of all cases. A real-world study published in 2016 stratified the mastectomy patients by radiotherapy receipt in Supplementary Tables, demonstrating better breast cancer-specific survival with receipt of BCS plus radiotherapy compared with mastectomy plus RT among stage II-III breast cancer [10]. In 2019, Lan XW et al. reviewed 1816 stage T1–2N1M0 female patients from Sun Y at Sen Memorial Hospital, supporting the results of Agarwal S et al. [19]. Yet, Sun GY et al. demonstrated survival equivalence between BCS plus RT group and mastectomy plus RT group by analyzing 4262 T1–2N1M0 patients in 2020 [20]. There was still no conclusion for breast cancer patients in the intermediate stage, which was the best choice to result in prolonged survival. These results may hardly be representative due to a relatively small number of cases and subgroup analysis results. De Boniface J. collected 48 986 T1–2 N0–2 Swedish data on breast cancer prospectively. Compared to mastectomy with or without radiation, 5-year OS and BCSS were significantly better after BCS. Numerous variables were controlled for in his study, including tumor characteristics, treatment, demographics, comorbidity, and socioeconomic background [21]. Our study is a retrospective analysis of a large cohort. We gathered data on all stage I-III American female breast cancer cases who have received BCS or mastectomy followed by postoperative RT from the SEER database. We include T3–4 and N3 populations in addition to T0–1N1–2, and the average follow-up time for the entire population is 79 months. We made a direct survival comparison based on stage and used PSM to equilibrate confounders. Our study was the first to identify which subgroups of patients benefit most from BCS plus RT and to develop a stage II patient prognostic nomogram.

The biological mechanism by which BCS plus RT increased survival remains unknown. A study by Onitilo AA et al. showed that BCS plus RT had a better survival rate than the mastectomy group. They concluded that both postoperative radiotherapy and the surgical method itself were likely to have contributed to such a significant survival difference [26]. The advantages of BCS plus RT in early breast cancer have been reported in the population of the United States [5,6], Canada [7], the Netherlands [10], China [27], and South Korea [28]. They tried to believe the advantages of BCS plus RT may be attributed partly to the effects of postoperative radiotherapy treatment. Likewise, a recent Chinese retrospective study corroborated the role of RT by grouping patients according to whether they had received RT, demonstrating the RT group had better 5-year DMFS, DFS, and BCSS compared with the no-RT group [19]. We believe that the improved survival noted in RT plus RT group is not only related to the surgical method but also closely related to the postoperative radiotherapy or interaction between radiotherapy and the immune microenvironment. As we all know, RT can regulate the tumor microenvironment in various ways, including regulating the release of local cytokines, chemokines, and other soluble factors, remodeling the structure of interstitial tumor cells and vascular cells, etc. [29,30,31,32,33]. Goodman CR et al. reported that postoperative radiotherapy prolonged the survival of circulating tumor cell-positive-positive patients undergoing BCS but did not prolong the survival of mastectomy [34]. This may indicate that the immune microenvironment can work with radiotherapy to improve the survival rate of BCS.

According to our results, stage II breast patients who received BCS plus RT had better survival compared with receiving mastectomy plus RT, and the results were consistent before and after matching. For patients with stage I and stage III, the survival of BCS plus RT seem to be similar to that of mastectomy plus RT. The reason for this phenomenon may be that the larger tumor burden and higher risk of metastasis in stage III patients limit the benefits provided by RT. Stage I patients are characterized by smaller tumor burden, breast-conserving surgery and mastectomy can create similar survival, and the synergistic effect of radiotherapy and immunity has not yet been exerted. In a subset of patients with stage 2A (T0–1N1, T2N0), the effect of BCS plus RT on OS and BCSS can be observed. However, the mechanism is still poorly understood, and we plan to explore the answer in the following research.

The limitation lies in that this study is a retrospective study, and the results of this study need to be proved by a robust prospective trial. Significant imbalances of certain covariates may be unavoidable in PSM. We regret that the PR status could not be perfectly matched, while other confounders presented no significant imbalance. Human epidermal growth factor receptor 2 (HER2) expression in breast cancer patients was only documented in the SEER database starting in 2010. Unfortunately, the follow-up time was insufficiently long to include HER2 in the study. In addition, the lack of data information on local recurrence and remote metastasis, the lack of Ki67, BRCA1, BRCA2 mutations, and other genetic information, and the unsubdivided chemotherapy situation are all the limitations of this study.

## 5. Conclusions

In summary, BCS plus RT had a superior treatment effect as that of mastectomy plus RT for stage II patients and also provided an equivalent survival for stage I or stage III patients. Moreover, the nomogram was a good predictor of 5-, 10-, and 15-year BCSS for stage II patients. Our results may help guide treatment decisions and prolong the survival of breast cancer patients.

## Figures and Tables

**Table 1:** Clinical characteristics of stage I–III patients.

	Overall	Mastectomy Plus RT	BCS Plus RT	*p*	Test
*n*	24,590	10,785	13,805		
Median follow-up time	79.00 [37.00, 135.00]	63.00 [29.00, 110.00]	95.00 [47.00, 149.00]	<0.001	nonnorm
Age_diagnosis					
(median [IQR])	57.00 [48.00, 66.75]	53.00 [45.00, 63.00]	59.00 [51.00, 68.00]	<0.001	nonnorm
Age (%)				<0.001	
<65	17,589 (71.5)	8476 (78.6)	9113 (66.0)		
>65	7001 (28.5)	2309 (21.4)	4692 (34.0)		
Race (%)				<0.001	
Black	2885 (11.7)	1390 (12.9)	1495 (10.8)		
White	19,766 (80.4)	8373 (77.6)	11,393 (82.5)		
Other	1939 (7.9)	1022 (9.5)	917 (6.6)		
Histologic_Type (%)				<0.001	
Duct carcinoma	18,092 (73.6)	7454 (69.1)	10,638 (77.1)		
Lobular carcinoma	2120 (8.6)	1262 (11.7)	858 (6.2)		
Other	4378 (17.8)	2069 (19.2)	2309 (16.7)		
Grade (%)				<0.001	
Grade I	4388 (17.8)	988 (9.2)	3400 (24.6)		
Grade II	10,743 (43.7)	4581 (42.5)	6162 (44.6)		
Grade III	9188 (37.4)	5070 (47.0)	4118 (29.8)		
Grade IV	271 (1.1)	146 (1.4)	125 (0.9)		
Laterality (%)				0.015	
Left	12,397 (50.4)	5520 (51.2)	6877 (49.8)		
right	12,187 (49.6)	5260 (48.8)	6927 (50.2)		
others	6 (0.0)	5 (0.0)	1 (0.0)		
Stage (%)				<0.001	
I	9264 (37.7)	691 (6.4)	8573 (62.1)		
II	8296 (33.7)	3916 (36.3)	4380 (31.7)		
III	7030 (28.6)	6178 (57.3)	852 (6.2)		
T (%)				<0.001	
T0	34 (0.1)	26 (0.2)	8 (0.1)		
T1	12,813 (52.1)	2475 (22.9)	10,338 (74.9)		
T2	7711 (31.4)	4586 (42.5)	3125 (22.6)		
T3	2680 (10.9)	2416 (22.4)	264 (1.9)		
T4	1352 (5.5)	1282 (11.9)	70 (0.5)		
N (%)				<0.001	
N0	12,041 (49.0)	1729 (16.0)	10,312 (74.7)		
N1	7180 (29.2)	4406 (40.9)	2774 (20.1)		
N2	3381 (13.7)	2885 (26.8)	496 (3.6)		
N3	1988 (8.1)	1765 (16.4)	223 (1.6)		
Intervention (%)				<0.001	
Mastectomy plus RT	10,785 (43.9)	10,785 (100.0)	0 (0.0)		
BCS plus RT	13,805 (56.1)	0 (0.0)	13,805 (100.0)		
Chemotherapy (%)				<0.001	
Yes	14,689 (59.7)	9198 (85.3)	5491 (39.8)		
No and unknown	9901 (40.3)	1587 (14.7)	8314 (60.2)		
Tumor_size (%)				<0.001	
<1cm	17,843 (72.6)	7389 (68.5)	10,454 (75.7)		
1–2 cm	3176 (12.9)	974 (9.0)	2202 (16.0)		
2–3 cm	1561 (6.3)	770 (7.1)	791 (5.7)		
3–4cm	665 (2.7)	467 (4.3)	198 (1.4)		
4–5 cm	427 (1.7)	347 (3.2)	80 (0.6)		
>5 cm	918 (3.7)	838 (7.8)	80 (0.6)		
Regional_nodes_positive					
(mean (SD))	2.47 (4.60)	4.70 (5.71)	0.72 (2.29)	<0.001	
ER_status (%)				<0.001	
Positive	19,769 (80.4)	8312 (77.1)	11,457 (83.0)		
Negative	4780 (19.4)	2455 (22.8)	2325 (16.8)		
Borderline	41 (0.2)	18 (0.2)	23 (0.2)		
PR_status (%)				<0.001	
Positive	17,237 (70.1)	7110 (65.9)	10,127 (73.4)		
Negative	7253 (29.5)	3632 (33.7)	3621 (26.2)		
Borderline	100 (0.4)	43 (0.4)	57 (0.4)		
Status				<0.001	
Alive	19,268 (78.4)	7791 (72.2)	11,477 (83.1)		
Dead	5322 (21.6)	2994 (27.8)	2328 (16.9)		

Variables included age, race, grade, laterality, stage, tumor size, lymph node infiltration, ER and PR status, chemotherapy, and treatment strategies (BCS plus RT vs. mastectomy plus RT).

**Table 2:** Clinical characteristics of patients for stage II before and after propensity score matching (PSM).

	PSM before			PSM after		
	Mastectomy Plus RT	BCS Plus RT	*p*	Mastectomy Plus RT	BCS Plus RT	*p*
*n*	3916	4380		2631	2631	
Age (%)			<0.001			0.433
<65	3184 (81.3)	3008 (68.7)		2035(77.3)	2010(76.4)	
>65	732 (18.7)	1372 (31.3)		596 (22.7)	621 (23.6)	
Race (%)			0.558			0.54
Black	498 (12.7)	577 (13.2)		352(13.4)	336(12.8)	
Non-black	3418 (87.3)	3803 (86.8)		2279 (86.6)	2295 (87.2)	
Grade (%)			0.111			0.824
well/moderately	2185 (55.8)	2521 (57.6)		1492(56.7)	1501(57.1)	
poorly differentiated	1731 (44.2)	1859 (42.4)		1139 (43.3)	1130 (42.9)	
T (%)			<0.001			0.477
T0	8 (0.2)	4 (0.1)		2 (0.1)	4 (0.2)	
T1	978 (25.0)	1472 (33.6)		939 (35.7)	985 (37.4)	
T2	2538 (64.8)	2771 (63.3)		1559 (59.3)	1512 (57.5)	
T3	392 (10.0)	133 (3.0)		131 (5.0)	130 4.9)	
N (%)			<0.001			0.165
N0	980 (25.0)	1850 (42.2)		2509(85.6)	2520(86.9)	
N1	2936 (75.0)	2530 (57.8)		122 (14.4)	111 (13.1)	
Chemotherapy (%)			<0.001			0.894
Yes	3269 (83.5)	2849 (65.0)		2063(78.4)	2058(78.2)	
No and unknown	647 (16.5)	1531 (35.0)		568 (21.6)	573 (21.8)	
Tumor_size (%)			<0.001			0.215
<1 cm	2814 (71.9)	2935 (67.0)		1863 (70.8)	1841 (70.0)	
1–2 cm	270 (6.9)	483 (11.0)		253 (9.6)	305 (11.6)	
2–3 cm	343 (8.8)	703 (16.1)		298 (11.3)	268 (10.2)	
3–4 cm	210 (5.4)	165 (3.8)		122 (4.6)	128 (4.9)	
4–5 cm	166 (4.2)	61 (1.4)		58 (2.2)	57 (2.2)	
>5 cm	113 (2.9)	33 (0.8)		37 (1.4)	32 (1.2)	
ER_status (%)			0.212			0.064
Positive	3129 (79.9)	3442 (78.6)		2075 (78.9)	2004 (76.2)	
Negative	782 (20.0)	935 (21.3)		554 (21.1)	625 (23.8)	
Borderline	5 (0.1)	3 (0.1)		2 (0.1)	2 (0.1)	
PR_status (%)			0.689			<0.001
Positive	2705 (69.1)	2999 (68.5)		1804 (68.6)	1596 (60.7)	
Negative	1200 (30.6)	1365 (31.2)		820 (31.2)	1024 (38.9)	
Borderline	11 (0.3)	16 (0.4)		7 (0.3)	11 (0.4)	

A 1: 1 ratio and the caliper of 0.02 was set. A total of 2631 pairs of patients were selected by PSM from the initial stage II data.

**Table 3:** Clinical characteristics of patients for stage III before and after propensity score matching (PSM).

	PSM before			PSM after		
	Mastectomy Plus RT	BCS Plus RT	*p*	Mastectomy Plus RT	BCS Plus RT	*p*
*n*	6178	852		852	852	
Age (%)			0.93			1
<65	4737 (76.7)	655 (76.9)		654 (76.8)	655 (76.9)	
>65	1441 (23.3)	197 (23.1)		198 (23.2)	197 (23.1)	
Race (%)			0.002			0.395
Black	824 (13.3)	147 (17.3)		133 (15.6)	147 (17.3)	
Non-black	5354 (86.7)	705 (82.7)		719 (84.4)	705 (82.7)	
Grade (%)			0.099			1
Well/moderately	2931 (47.4)	378 (44.4)		377 (44.2)	378 (44.4)	
Differentiated						
Poorly differentiated/	3247 (52.6)	474 (55.6)		475 (55.8)	474 (55.6)	
Undifferentiated						
T(%)			<0.001			0.964
T0	17 (0.3)	3 (0.4)		3 (0.4)	3 (0.4)	
T1	807 (13.1)	294 (34.5)		305 (35.8)	294 (34.5)	
T2	2048 (33.1)	354 (41.5)		344 (40.4)	354 (41.5)	
T3	2024 (32.8)	131 (15.4)		135 (15.8)	131 (15.4)	
T4	1282 (20.8)	70 (8.2)		65 (7.6)	70 (8.2)	
N (%)			<0.001			0.958
N0	140 (2.3)	22 (2.6)		19 (2.2)	22 (2.6)	
N1	1388 (22.5)	111 (13.0)		114 (13.4)	111 (13.0)	
N2	2885 (46.7)	496 (58.2)		500 (58.7)	496 (58.2)	
N3	1765 (28.6)	223 (26.2)		219 (25.7)	223 (26.2)	
chemotherapy (%)			0.082			0.361
Yes	5548 (89.8)	748 (87.8)		761 (89.3)	748 (87.8)	
No and unknown	630 (10.2)	104 (12.2)		91 (10.7)	104 (12.2)	
Tumor_size (%)			<0.001			0.875
<1 cm	4014 (65.0)	542 (63.6)		541 (63.5)	542 (63.6)	
1–2 cm	577 (9.3)	128 (15.0)		136 (16.0)	128 (15.0)	
2–3 cm	427 (6.9)	85 (10.0)		72 (8.5)	85 (10.0)	
3–4 cm	257 (4.2)	31 (3.6)		30 (3.5)	31 (3.6)	
4–5 cm	181 (2.9)	19 (2.2)		23 (2.7)	19 (2.2)	
>5 cm	722 (11.7)	47 (5.5)		50 (5.9)	47 (5.5)	
ER_status (%)			0.625			0.555
Positive	4613 (74.7)	624 (73.2)		617 (72.4)	624 (73.2)	
Negative	1555 (25.2)	227 (26.6)		235 (27.6)	227 (26.6)	
Borderline	10 (0.2)	1 (0.1)		0 (0.0)	1 (0.1)	
PR_status (%)			0.76			0.13
Positive	3909 (63.3)	528 (62.0)		536 (62.9)	528 (62.0)	
Negative	2240 (36.3)	320 (37.6)		316 (37.1)	320 (37.6)	
Borderline	29 (0.5)	4 (0.5)		0 (0.0)	4 (0.5)	

A 1: 1 ratio was set. A total of 852 pairs of patients were selected by PSM from the original stage III patients.

**Table 4:** Univariate and multivariate analysis of OS for stage II patients.

Variables	Univariable Cox		Multivariable Cox	
	HR	*p*	HR	*p*
Intervention				
Mastectomy plus RT	control group		control group	
BCS plus RT	0.6893(0.5957–0.7977)	<0.001	0.6730(0.5754–0.787)	<0.001
Age				
<65	control group		control group	
>65	1.333(1.132–1.57)	<0.001	1.6659(1.4093–1.969)	<0.001
Race				
Black	control group		control group	
Non-black	0.5986(0.4939–0.7255)	<0.001	0.7257(0.5965–0.883)	0.0014
Grade				
I/II	control group		control group	
III/IV	2.364(2.032–2.75)	<0.001	1.9366(1.6396–2.287)	<0.001
N				
N0	control group		control group	
N1	1.212(1.033–1.421)	0.0181	1.4052(1.1801–1.673)	<0.001
Tumor_size				
<1 cm	control group		control group	
1–2 cm	1.003(0.7865–1.278)	0.982	1.0748(0.8361–1.382)	0.5737
2–3 cm	1.692(1.3974–2.048)	<0.001	1.7675(1.4528–2.150)	<0.001
3–4 cm	2.089(1.6061–2.717)	<0.001	1.8681(1.4330–2.435)	<0.001
4–5 cm	1.371(0.9250–2.031)	0.116	1.1356(0.7626–1.691)	0.5313
>5 cm	1.016(0.5563–1.856)	0.959	0.9930(0.5339–1.847)	0.9822
ER_status				
Positive	control group		control group	
Negetive	2.1286(1.8272–2.480)	<0.001	1.2908(1.0317–1.615)	0.0255
Borderline	0.7376(0.1034–5.262)	0.761	0.6434(0.0880–4.705)	0.664
PR_status				
Positive	control group		control group	
Negetive	2.045(1.7672–2.367)	<0.001	1.3798(1.1202–1.700)	0.0025
Borderline	1.771(0.6613–4.745)	0.255	1.3948(0.5136–3.788)	0.5139
T				
T0	control group			
T1	305,014.32	0.985		
T2	538,153.28	0.984		
T3	457,474.89	0.985		
chemotherapy				
YES	control group			
NO	0.9175(0.7711–1.092)	0.331		

## Data Availability

The datasets analyzed during the current study are available from the National Cancer Institute’s Surveillance, Epidemiology, and End Results program database: https://seer.cancer.gov/data/access.html (accessed on 25 June 2020).

## Supplementary Materials

The following supporting information can be downloaded at: https://www.mdpi.com/article/10.3390/curroncol29080452/s1, Supplementary Table S1: Univariate and multivariate analysis of OS for I-III patients; Supplementary Table S2: Univariate and multivariate analysis of BCSS for I-III patients; Supplementary Figure S1: Subgroup analysis for OS and BCSS for stage III patients.

## Abbreviations

BCSS	Breast cancer-specific survival
BCS	Breast-conserving surgery
RT	Radiotherapy
OS	Overall survival
PSM	Propensity score matching
ER	Estrogen receptor
PR	Progesterone receptor
HER2	Human epidermal growth factor receptor 2
