# Supplementary material for: Survival Comparisons between Breast Conservation Surgery and Mastectomy Followed by Postoperative Radiotherapy in Stage I–III Breast Cancer Patients: Analysis of the Surveillance, Epidemiology, and End Results (Seer) Program Database
Source: Curr Oncol. 2022 Aug 15;29(8):5731–47. doi: 10.3390/curroncol29080452 (PMC9406949; doi:10.3390/curroncol29080452)
Supplement: Supplementary file 1 [file curroncol-29-00452-s001.zip › curroncol-1815302-supplementary.pdf]

Current Oncology

Article

Survival Comparisons between Breast Conservation Surgery and Mastectomy Followed by Postoperative Radiotherapy in Stage I-III Breast Cancer Patients: Analysis of the Surveillance, Epidemiology, and End Results (Seer) Program Database

Wenbin Xiang, Chaoyan Wu, Huachao Wu, Sha Fang, Nuomin Liu and Haijun Yu

Supplementary Table S1. Univariate and multivariate analysis of OS for I-III patients.

Variables	Univariable Cox		Multivariable Cox	
	HR	P	HR	P
age				
<65	control group		control group	
>65	2.317(2.194-2.447)	<0.001	2.6248(2.4718-2.7872)	<0.001
race				
black	control group		control group	
white	0.6082(0.5640-0.6559)	<0.001	0.6841(0.6336-0.7387)	<0.001
other	0.5498(0.4833-0.6254)	<0.001	0.6160(0.5412- 0.7012)	<0.001
Histologic_Type				
duct carcinoma	control group		control group	
Lobular carcinoma	1.1647(1.0569- 1.2840)	0.002	1.0470(0.9449-1.1601)	0.38
other	0.9674(0.9007-1.0390)	0.362	0.9500(0.8837-1.0212)	0.164
Grade				
I	control group		control group	
II	1.484 (1.352-1.629)	<0.001	1.2037(1.0943-1.3241)	<0.001
III	2.568(2.346 -2.811)	<0.001	1.6317(1.4759- 1.8041)	<0.001
IV	2.458(2.002-3.018)	<0.001	1.5926(1.2915-1.9640)	<0.001
Laterality				
left	control group		control group	
right	0.9607(0.9105-1.014)	0.144	0.9923(0.9403-1.0472)	0.779
others	1.8425(0.4606-7.371)	0.388	3.1303(0.7652-12.8055)	0.112
Stage				
I	control group		control group	
II	1.743 (1.617-1.878)	<0.001	1.1380(0.9991-1.2962)	0.052
III	4.046(3.777 -4.334)	<0.001	1.2690(1.0446-1.5417)	0.016
T				
T0	control group		control group	
T1	0.6077(0.2526-1.462)	0.266	1.2525(0.5120-3.0641)	0.622
T2	1.2425(0.5165-2.989)	0.628	1.9178(0.7851-4.6848)	0.153
T3	1.7341(0.7197-4.178)	0.22	2.3919(0.9762-5.8604)	0.056
T4	3.2003(1.3272-7.717)	0.01	3.1676(1.2932-7.7591)	0.012
N				
N0	control group		control group	
N1	1.787(1.667 -1.915)	<0.001	1.5820(1.4227-1.7591)	<0.001
N2	3.010(2.794 -3.243)	<0.001	2.2036(1.8782-2.5855)	<0.001

N3	4.851(4.477-5.257)	<0.001	3.2458(2.7680-3.8060)	<0.001
intervention				
RT after mastectomy	control group		control group	
RT after BCS	0.4335(0.4105-0.4578)	<0.001	0.8473(0.7841-0.9155)	<0.001
chemotherapy				
YES	control group		control group	
NO	0.8305(0.7858-0.8777)	<0.001	1.6588(1.5423-1.7840)	<0.001
Tumor_size				
<1cm	control group		control group	
1-2cm	1.142(1.060- 1.231)	<0.001	1.4129(1.3024 - 1.5328)	<0.001
2-3cm	1.647(1.505-1.802)	<0.001	0.9435(0.9597-1.1798)	0.239
3-4cm	2.278(2.015-2.575)	<0.001	1.3007(1.1409-1.4830)	<0.001
4-5cm	2.156(1.852-2.510)	<0.001	1.2767(1.0889-1.4968)	0.003
>5cm	2.710(2.443-3.006)	<0.001	1.0653(0.9410-1.2059)	0.318
ER_status				
positive	control group		control group	
Negetive	1.794(1.691- 1.904)	<0.001	1.3726(1.2620-1.4930)	<0.001
Borderline	1.259(0.745-2.129)	0.389	0.7407(0.4355-1.2597)	0.268
PR_status				
positive	control group		control group	
Negetive	1.712(1.621-1.809)	<0.001	1.2145(1.1265-1.3094)	<0.001
Borderline	1.908(1.402-2.596)	<0.001	1.5524(1.1374 -2.1187)	0.006

Supplementary Table S2. Univariate and multivariate analysis of BCSS for I-III patients.

Variables	Univariable Cox		Multivariable Cox	
	HR	P	HR	P
age				
<65	control group		control group	
>65	1.137(1.055-1.225)	0.001	1.532(1.412-1.662)	<0.001
race				
black	control group		control group	
white	0.547(0.500-0.598)	<0.001	0.695(0.634-0.761)	<0.001
other	0.563(0.484-0.655)	<0.001	0.635(0.545-0.739)	<0.001
Histologic_Type				
duct carcinoma	control group		control group	
Lobular carcinoma	1.058(0.935-1.197)	0.374	1.104(0.968-1.258)	0.141
other	0.921(0.841-1.007)	0.072	0.948(0.865-1.039)	0.251
Grade				
I	control group		control group	
II	2.607(2.229-3.048)	<0.001	1.633(1.394-1.915)	<0.001
III	6.007(5.164-6.988)	<0.001	2.383(2.028-2.801)	<0.001
IV	6.042(4.639-7.868)	<0.001	2.498(1.907-3.271)	<0.001
Laterality				
left	control group		control group	
right	0.939(0.878-1.004)	0.064	0.974(0.911-1.042)	0.443
others	2.875(0.719-11.506)	0.136	5.342(1.282-22.261)	0.021
Stage				
I	control group		control group	
II	3.897(3.444-4.409)	<0.001	1.780(1.482-2.136)	<0.001
III	11.289(10.056-12.672)	<0.001	2.108(1.649-2.696)	<0.001

T				
T0	control group		control group	
T1	0.496(0.160-1.542)	0.226	2.057(0.640-6.614)	0.226
T2	1.670(0.538-5.184)	0.375	3.119(0.972-10.013)	0.056
T3	2.514(0.809-7.814)	0.111	3.908(1.214-12.583)	0.022
T4	4.221(1.356-13.135)	0.013	4.784(1.486-15.397)	0.009
N				
N0	control group		control group	
N1	3.193(2.895-3.522)	<0.001	1.694(1.481-1.937)	<0.001
N2	6.124(5.538-6.772)	<0.001	2.429(2.008-2.939)	<0.001
N3	10.726(9.672-11.896)	<0.001	3.842(3.182-4.640)	<0.001
intervention				
RT after mastectomy	control group		control group	
RT after BCS	0.263(0.244-0.283)	<0.001	0.820(0.746-0.901)	<0.001
chemotherapy				
YES	control group		control group	
NO	0.372(0.343-0.404)	<0.001	1.267(1.146-1.401)	<0.001
Tumor_size				
<1cm	control group		control group	
1-2cm	1.187(1.076-1.311)	<0.001	1.560(1.398-1.741)	<0.001
2-3cm	2.152(1.932-2.399)	<0.001	1.243(1.102-1.403)	<0.001
3-4cm	3.346(2.912-3.844)	<0.001	1.619(1.397-1.875)	<0.001
4-5cm	2.937(2.463-3.502)	<0.001	1.492(1.243-1.791)	<0.001
>5cm	3.828(3.397-4.314)	<0.001	1.240(1.078-1.426)	0.003
ER_status				
positive	control group		control group	
Negetive	2.442(2.276-2.619)	<0.001	1.448(1.309-1.602)	<0.001
Borderline	1.716(0.922-3.194)	0.089	0.997(0.532-1.869)	0.992
PR_status				
positive	control group		control group	
Negetive	2.227(2.081-2.382)	<0.001	1.312(1.194-1.442)	<0.001
Borderline	2.331(1.605-3.386)	<0.001	1.865(1.279-2.719)	0.001

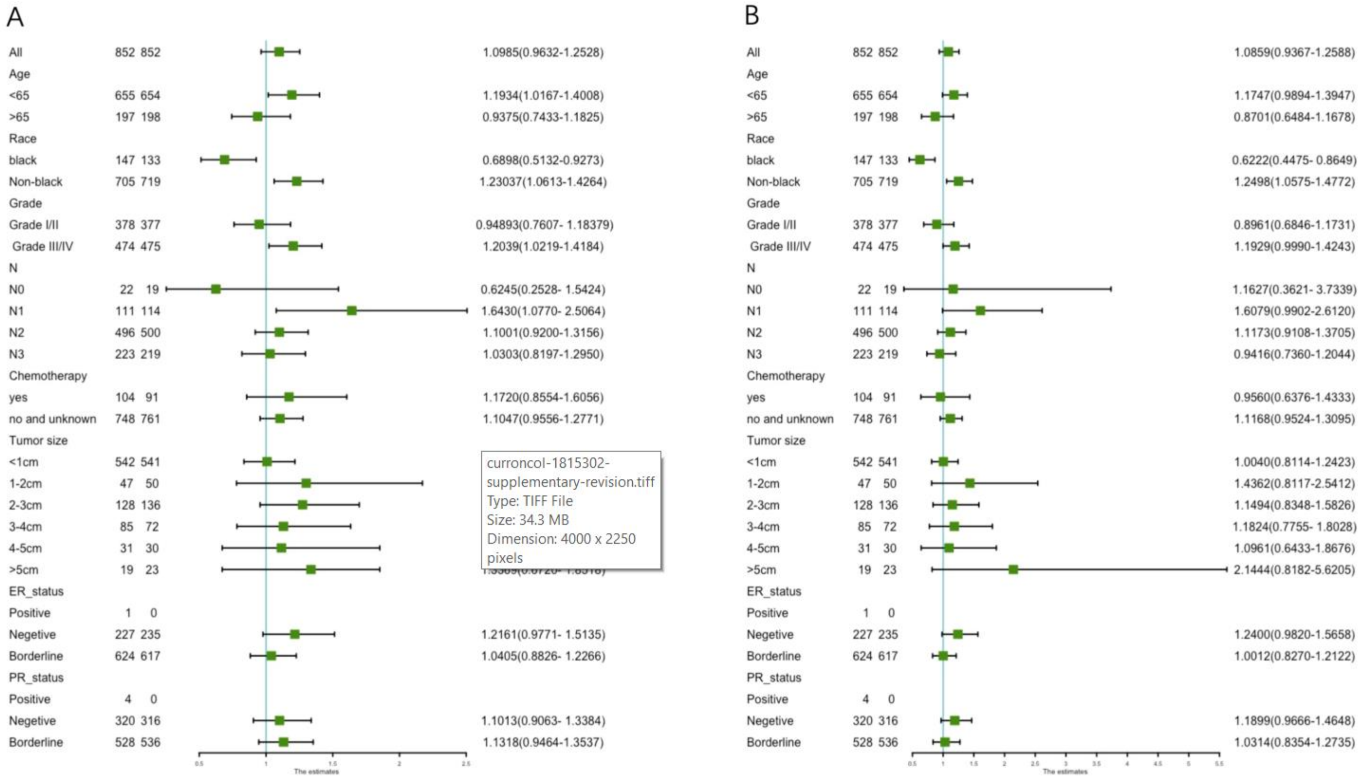

Supplementary Figure S1. Subgroup analysis for OS and BCSS for stage III patients. (A) Forest map for OS. (B) Forest map for BCSS.
